# Characterization of Simultaneous Pressure Waves as Biomarkers for Colonic Motility Assessed by High-Resolution Colonic Manometry

**DOI:** 10.3389/fphys.2018.01248

**Published:** 2018-09-20

**Authors:** Ji-Hong Chen, Sean P. Parsons, Mitra Shokrollahi, Andrew Wan, Alexander D. Vincent, Yuhong Yuan, Maham Pervez, Wu Lan Chen, Mai Xue, Kailai K. Zhang, Arshia Eshtiaghi, David Armstrong, Premsyl Bercik, Paul Moayyedi, Eric Greenwald, Elyanne M. Ratcliffe, Jan D. Huizinga

**Affiliations:** ^1^Division of Gastroenterology, Department of Medicine, Farncombe Family Digestive Health Research Institute, McMaster University, Hamilton, ON, Canada; ^2^Sun Yat-sen University, Guangdong, China; ^3^Department of Pediatrics, McMaster University, Hamilton, ON, Canada

**Keywords:** colonic motility, anal sphincter pressure, simultaneous pressure waves, high-resolution manometry, gastro-colonic reflex, bisacodyl, high-amplitude propagating pressure wave

## Abstract

Simultaneous pressure waves (SPWs) in manometry recordings of the human colon have been associated with gas expulsion. Our hypothesis was that the SPW might be a critical component of most colonic motor functions, and hence might act as a biomarker for healthy colon motility. To that end, we performed high-resolution colonic manometry (HRCM), for the first time using an 84-sensor (1 cm spaced) water-perfused catheter, in 17 healthy volunteers. Intraluminal pressure patterns were recorded during baseline, proximal and rectal balloon distention, after a meal and following proximal and rectal luminal bisacodyl administration. Quantification was performed using software, based on Image J, developed during this study. Gas expulsion was always associated with SPWs, furthermore, SPWs were associated with water or balloon expulsion. SPWs were prominently emerging at the termination of proximal high amplitude propagating pressure waves (HAPWs); we termed this motor pattern HAPW-SPWs; hence, SPWs were often not a pan-colonic event. SPWs and HAPW-SPWs were observed at baseline with SPW amplitudes of 12.0 ± 8.5 mmHg and 20.2 ± 7.2 mmHg respectively. The SPW occurrence and amplitude significantly increased in response to meal, balloon distention and luminal bisacodyl, associated with 50.3% anal sphincter relaxation at baseline, which significantly increased to 59.0% after a meal, and 69.1% after bisacodyl. Often, full relaxation was achieved. The SPWs associated with gas expulsion had a significantly higher amplitude compared to SPWs without gas expulsion. SPWs could be seen to consist of clusters of high frequency pressure waves, likely associated with a cluster of fast propagating, circular muscle contractions. SPWs were occasionally observed in a highly rhythmic pattern at 1.8 ± 1.2 cycles/min. Unlike HAPWs, the SPWs did not obliterate haustral boundaries thereby explaining how gas can be expelled while solid content can remain restrained by the haustral boundaries. In conclusion, the SPW may become a biomarker for normal gas transit, the gastrocolonic reflex and extrinsic neural reflexes. The SPW assessment reveals coordination of activities in the colon, rectum and anal sphincters. SPWs may become of diagnostic value in patients with colonic dysmotility.

## Introduction

Patients with functional bowel disorders have abnormal bowel movements and/or outlet obstruction and often complain about bloating (Burri et al., 2014; Malagelada et al., 2017). Some surveys show that abdominal bloating is the most bothersome symptom (Kanazawa et al., 2016). Patients with bloating have normal colonic accommodation of gas loads and ileocecal continence but impaired clearance of gas from the colon (Hernando-Harder et al., 2010). Patients with poor handling of gas, also show poor handling of semi–liquid contents (Serra et al., 2010). Impaired gas clearance appears to involve tonic contractions of the small intestine (Tremolaterra et al., 2006; Serra et al., 2010) and abnormal motor activity of the colon (Malagelada et al., 2017). Proof of concept studies show a positive effect of prokinetics on gas related symptoms (Serra et al., 2001; Caldarella et al., 2002) which suggests that gut motor activity determines gas transit, and that bloating is caused by a motility disorder; however, the precise mechanisms underlying gas transit have not been established.

Clinical colonic manometry studies focus on bisacodyl- and/or meal-induced motor patterns (Camilleri et al., 2008; Rao et al., 2011; Mugie et al., 2013; Dinning et al., 2016), in particular high amplitude propagating pressure waves (HAPWs, De Schryver et al., 2003; Chen et al., 2017), also called high amplitude contractions (HAPCs, Bassotti and Gaburri, 1988) or high amplitude propagating sequences (Dinning et al., 2004), but there has been little or no emphasis on the study of specific gas-associated motor patterns. Clinical colonic manometry is mostly focused on the identification of a potential “inert” colon, usually defined as the absence of HAPWs during a colon function test, and when thus diagnosed, surgery is contemplated. There are, however, many uncertainties related to such a conclusion. First, healthy volunteers may not have HAPWs in a 24 h period (Cook et al., 2000; Rao et al., 2001; Dinning et al., 2010), hence whether absence of HAPW definitely indicates an abnormal colon is questionable. It is therefore also not clear if slow transit is explained by an absence of HAPWs (Bharucha, 2012). It is also uncertain if the colon can be deemed normal if HAPWs are induced only by bisacodyl, since bisacodyl may induce HAPWs whereas physiological stimuli may not (Liem et al., 2010; Bharucha, 2012). These uncertainties, and the understanding that colonic motility often is analyzed in a cursory manner, may be a reason why colonic motility tests are not often performed in adults; and in children they add uncertainty to the outcome. Hence, if a colonic motility test is to assess the full capabilities of the colon, including motor patterns associated with gas evacuation, it needs to expand its scope. This possibility seems at the horizon with the application of high-resolution manometry to the colon. Dinning showed the marked increase in understanding of motor patterns with high resolution manometry and the danger of interpreting motor patterns using low-resolution manometry (Dinning et al., 2013). The present study focuses on the gas-expulsion-related colonic motor pattern, the SPW. SPWs are pressure transients that occur simultaneously at all sensors that record them. Rao et al. (2001) identified the SPW as a common motor pattern but it was largely ignored in subsequent studies due to the uncertainty about it being related to abdominal pressure changes. We recently reported on SPWs using high-resolution manometry with a solid-state catheter incorporating 36 sensors at 1 cm spacing covering the descending colon down to the anal sphincter (Chen et al., 2017). The term “simultaneous contraction” was avoided since contraction was not measured and pressure is not equivalent to contraction (Chen et al., 2016; Quan et al., 2017). Corsetti et al. (2017) reported the phenomenon using a catheter with 40 solid state sensors at 2.5 cm spacing, calling them pan-colonic pressurizations.

The present study characterized SPWs for the first time using high-resolution manometry with 84 sensors at 1 cm spacing throughout the entire colon including the anal sphincters. In order to fully understand the physiological importance of SPWs, we designed a protocol with the following stimuli: a 1000 kcal fat-rich meal, luminal balloon distention and luminal bisacodyl administration. Furthermore, we present the colonic motor patterns in three-dimensional format to facilitate visual interpretation.

The National Institutes of Health Biomarkers Definitions Working Group defined a biomarker as “a characteristic that is objectively measured and evaluated as an indicator of *normal biological processes*, pathogenic processes, *or pharmacologic responses* to a therapeutic intervention” (Biomarkers, 2001). Here we characterize the SPW as just this kind of biomarker, starting with what role it plays under normal physiological conditions and in response to pharmacological substances in healthy subjects. This comprehensive characterization of SPWs in healthy subjects by HRCM may provide significant clinical value to evaluate pathophysiology in patients with colonic dysmotility and abdominal bloating.

## Materials and Methods

### Study Subjects

Seventeen healthy subjects (age: 20–54 years; 6 females) were recruited as volunteers through local advertising. All participants gave written informed consent and all procedures were approved by the Hamilton Integrated Research Ethics Board (HiREB). Exclusion criteria were: abdominal surgery, hepatic, kidney or cardiac diseases, connective tissue disorders, central nervous system disorders, thyroid diseases, prostate diseases or malignancies. All subjects had normal stool consistency and normal bowel frequency, between 1 every 3 days and 3 per day. None had defecation difficulty, and none were taking medication that might influence bowel movements.

### High-Resolution Colonic Manometry

High-resolution colonic manometry was performed on a custom-made platform [Medical Measurement Systems (MMS); Laborie, Toronto, ON, Canada). An 84-sensor water-perfused catheter was designed (Mui Scientific, Mississauga, ON, Canada) that included two 10-cm long balloons between sensors 10 and 11 and 40 and 41, with sensor 1 placed in the ascending colon. In four volunteers, a separate rectal balloon was used. The catheter was inserted with minimal sedation (fentanyl i.v. 50–100 mcg and midazolam i.v. 2 – 5 mg) with the assistance of a colonoscope after a bowel cleaning procedure using an inert osmotic laxative (PEG-Lyte, Pendopharm, Montreal, QC, Canada), but no use of stimulant laxatives such as bisacodyl. For the bowel cleaning procedure, 3 L of PEG (70 g/L) was taken between 4 and 6 pm the day before the procedure, with more water consumed if needed to have all solids removed. The next morning, 1 L was taken at 4 am. The tip of the catheter was clipped to the mucosa via a fish line, a few cm distal to the cecum. The catheter was made of 100% silicon; after use, an extensive approved cleaning procedure was executed followed by sterilization. A disposable dual lumen stomach tube (3.3 mm × 91 cm; Salem Sump^TM^, Covidien Ilc, United States) was placed in the rectum for passive liquid drainage. All subjects were in the supine position during the entire recording, except during intake of the meal. The subjects were instructed to report all events such as gas or liquid expulsion, bowel movements, pain, and discomfort. The subjects were asked not to promote or prevent gas or liquid expulsion by increasing abdominal pressure or contracting the external anal sphincter if an urge arose, but to let their colonic motor activity allow events to occur without intervention. All body movements such as changing body position, talking, coughing, laughing, and urination were noted immediately into the data acquisition files.

### Protocol

A 90 min recording of baseline activity was started 30 min after the colonoscope was withdrawn. Thereafter, the response to a 5 min balloon distention at the proximal colon was investigated. Distal balloon distention induced activity is not reported here. The proximal balloon was distended with 250 – 400 ml air while the severity of abdominal discomfort reached to 6–7 on a 10-point symptom scale, then responses were recorded during and 20 min after the deflation of the balloon. Thereafter, a meal was given (500 g of organic vanilla yogurt fortified with organic milk fat (Mapleton Organics, Moorefield, ON, Canada), to reach 800 – 1000 kcal depending on the volume consumed). Its effect was observed for a minimum of 90 min. Lastly, the effects of 10 mg of bisacodyl (Dulcolax; Boehringer Ingelheim, Sanofi Canada, Laval, QC, Canada) in the proximal colon via the catheter or per rectum were observed for 30 min; the bisacodyl suspension was made in saline by crushing 2 tablets, 5 mg each, with a pestle and mortar for 5 min.

### Water-Perfusion

The catheter delivered 0.1 mL/min sterile water via each sensor to a total of 0.57 L per hour; the water pressure was calibrated at 1000 mbar. A drainage tube (OD = 3.3 mm) was placed in the rectum to allow passive outflow of excess water. In this way, 1–2 L water was diverted without notice by the subjects; the remainder of the water was expelled by colon motor activities or absorbed. Intraluminal pressure in between motor patterns did not change during the 6–8-h session, hence the water inflow did not cause passive tonic pressure changes.

#### Identified Motor Patterns in the Present Study

(1) Simultaneous pressure waves (SPWs) are pressure transients that occur simultaneously at all sensors that record them, as identified by Rao et al. (2001) and De Schryver et al. (2003), and further defined by us (Chen et al., 2014, 2017). They have also been called simultaneous contractions (Rao et al., 2004) or pan-colonic pressurizations (Corsetti et al., 2017). The term contraction was avoided since contraction was not measured and pressure is not equivalent to contraction (Chen et al., 2016; Quan et al., 2017).

(2) High amplitude propagating pressure waves. HAPWs are defined as transient increases in pressure of > 75 mmHg that propagate, almost always in anal direction. They are also called high amplitude propagating contractions or sequences (Bharucha, 2012).

(3) High amplitude propagating pressure waves followed by simultaneous pressure waves (HAPW-SPWs). This frequently occurring motor pattern is described in the present manuscript. An HAPW starts in the proximal colon and promptly switched to an SPW at the transverse or descending colon; hence the SPW within this motor pattern is not pan-colonic.

(4) Haustral boundaries are rhythmic or continuous pressure increases that occur in single sensors without activity in adjacent sensors; they occurred in multiple sensors about 5 cm apart (Chen et al., 2017; Quan et al., 2017). These pressure increases define the boundaries of haustra (Bharucha and Brookes, 2012).

(5) Haustral activity was an activity that occurred in 4–5 consecutive sensors but not immediately outside these sensors, suggesting it to be activity within a haustrum (Chen et al., 2017; Quan et al., 2017).

(6) Anal sphincter activity and anal sphincter relaxation, either spontaneously or as part of a motor pattern.

### Visual Identification and Quantitative Analysis of Motor Patterns

All data were aquired using the software developed by Medical Measurement Systems (MMS^®^; Laborie, Toronto, ON, Canada), and analyzed using programs developed by us using Image J^®^ (National Institutes of Health, Bethesda, MD, United States) and Matlab^®^ (Mathworks, Natick, MA, United States). After a motor pattern was identified by eye in Image J, a rectangle was placed around the SPW, or a HAPW was encircled free hand. In high-resolution manometry, with an acquisition rate of 10/s, an average SPW has 10.080 data points when all sensors record it. A typical HAPW has many more. These data were used to calculate the average amplitude, duration and length in Image J. Matlab was used to generate the 3D images. This method is substantially different from measurements using low-resolution manometry where only a few points along the motor pattern are taken into account. This also indicates that normal values have to be re-evaluated in HRCM when compared to low resolution manometry.

A positive gastrocolic reflex was defined as an increase in motor patterns, compared to baseline in response to a meal (Snape et al., 1980).

### Analysis of Anal Sphincter Activities

When voluntary external anal sphincter contractions occurred, it was not possible to quantify relaxation. When the internal anal sphincter showed rhythmic pressure activity, and the SPW coincided with the “relaxation phase” of the rhythmicity, the relaxation induced by the SPW could not be assessed accurately. Because of the oscillatory nature of the anal sphincter pressure, relaxation was quantified when it reached > 25% of the average pressure value recorded in a 3 min period before the relaxation. The period of relaxation started with the first sign of relaxation and ended with return to its baseline pressure. In the middle of this period, complete anal sphincter relaxation could be achieved.

### Symptom Correlations and Artifacts

Subjects were instructed to report all symptoms, such as abdominal pain, passing gas or liquids per rectum, abdominal bloating, rectal urgency, urinary urgency. They were also instructed not to withhold or resist gas expulsion or liquid outflow. Artifacts were caused by body movement or coughing. Talking without body movement did not cause artifacts. Such artifacts were immediately written in the acquisition files and excluded from the analysis.

### Statistical Analysis

The present study was designed to record the baseline colonic motor activity, which was followed by sessions with different stimuli. It is a descriptive study to document the features of normal SPW activity in healthy volunteers. The stimuli were given consecutively, therefore, the responses to stimuli may have been influenced by the remaining activity of the previous stimulus, with the exception of the first stimulation (proximal balloon distention). The responses to stimuli were described and compared to baseline activity qualitatively and quantitatively. Data are given as mean ± SD. Significance was determined by one-way ANOVA with Dunnett’s post-test using Prism 7 software (GraphPad, United States), *P* < 0.05 was considered significant. For SPWs with and without gas/liquid expulsion and potential differences between male and female subjects, the statistical significance was determined using *t*-test (Prism 7).

### Effect of Attached Balloons on Spatiotemporal Maps

The position of the balloons in all figures is identified by a white line. The white line represents a gap of 10 cm where no data were recorded. The length of the colon covered by the sensors as indicated in the figures is the true length, with the balloon sections taken into account.

## Results

Simultaneous pressure waves were studied during baseline, in response to balloon distention, a meal and luminal bisacodyl.

### Two Dominant Types of SPWs Occur With Associated Anal Sphincter Activities

At baseline, numerous SPWs appeared along the entire length of the colon with an average amplitude of 12.1 ± 8.5 mmHg, ranging from 5 to 38.4 mmHg (**Table 1**). Their duration ranged from 2 to 58 s, average 13.8 ± 9.1 s (**Table 1**).

**Table 1:** Quantification of SPW activity in 17 healthy subjects.

	Total SPWs	SPWs with gas expulsion	HAPWs	HAPW-SPWs (HAPW data)	HAPW-SPWs (SPW data)
Baseline (90 min)					

Occurrence	*N* = 17	*N* = 8	*N* = 5	*N* = 7	*N* = 7
	*n* = 245	*n* = 15	*n* = 15	*n* = 37	*n* = 37
Amplitude (mmHg)	12.1 ± 7.3	20.8 ± 4.3^ooo^	146.6 ± 49.4	105.4 ± 49.7	20.21 ±10.2°
	5 – 38.4	7.1 – 31.0	74.8 – 270.9	34.3 – 206.2	2.9 – 38.4
Duration (seconds)	13.8 ± 9.1	10.8 ± 4.6	78.6 ± 30.2	52.4 ± 21.7	18.0 ± 12.3
	2 – 57.9	6 – 25	37 – 78	16 – 120	4.7 – 37.9

Proximal Balloon (20 min)					

Occurrence	*N* = 13	*N* = 5	*N* = 7	*N* = 11	*N* = 11
	*n* = 42	*n* = 15	*n* = 21	*n* = 20	*n* = 20
Amplitude (mmHg)	15.3 ± 7.4	26.6 ± 10.1^oooo^	150.5 ± 36.2	105.3 ± 63.8	15.5 ± 8.0
	5 – 49.6	9.6 – 45.0	95.3 – 203.8	27.4 – 236.9	3.9 – 32.0
Duration (seconds)	10.3 ± 8.0	8.1 ± 2.5	98.1 ± 46.9	67.8 ± 28.2^∗^	12.0 ± 7.7
	2 – 36	3 – 12	44 – 219	24.2 – 124	5 – 30.1

Meal (90 min)					

Occurrence	*N* = 17	*N* = 9	*N* = 5	*N* = 10	*N* = 10
	*n* = 282	*n* = 34	*n* = 7	*n* = 36	*n* = 36
Amplitude (mmHg)	15.3 ± 8.4	29.4 ± 12.6^∗^/^oooo^	154.5 ± 69.8	95.6 ± 35	25.2 ± 13.4
	5.1 – 47.2	2.1 – 66.3	63.8 – 146.0	31.3 – 176.5	6.7 – 61.3
Duration (seconds)	10.8 ± 7.1^∗∗∗∗^	9.3 ± 6.8	67.2 ± 24.9	45.4 ± 23.3	15.6 ± 8.1
	2 – 57.3	2 – 40	46 – 106	12 – 96	4 – 35

Bisacodyl (30 min)					

Occurrence	*N* ± 15	*N* = 8	*N* = 12	*N* = 13	*N* = 13
	*n* = 219	*n* = 12	*n* = 37	*n* = 73	*n* = 73
Amplitude (mmHg)	19.7 ± 9.9^∗∗∗∗^	29.0 ± 6.0^∗/ooo^	284.4 ± 50.9	132.3 ± 50.9	21.6 ± 13.1
	3 – 57.5	18.4 – 36.8	88.8 – 322.5	47.2 – 225.6	5 – 57.5
Duration (seconds)	10.1 ± 9.3^∗∗∗^	13.1 ± 4.7	77.6 ± 38.6	67.8 ± 37.3^∗^	15.1 ± 11.2
	2 – 57	4 – 20	15 – 168	14 – 216	2 – 57


*Data are expressed as average ± SD and range; except occurrence; (N = # of subjects; n, number of motor patterns). ^∗^Comparison with their baseline conditions: ^∗^P ≤ 0.05, ^∗∗^P ≤ 0.01, ^∗∗∗^P ≤ 0.001, ^∗∗∗∗^P ≤ 0.0001. ^o^Comparison between SPW with gas expulsion vs. SPW without gas expulsion; ^ooo^P = 0.0001,^oooo^P < 0.0001.*

**FIGURE 1:**
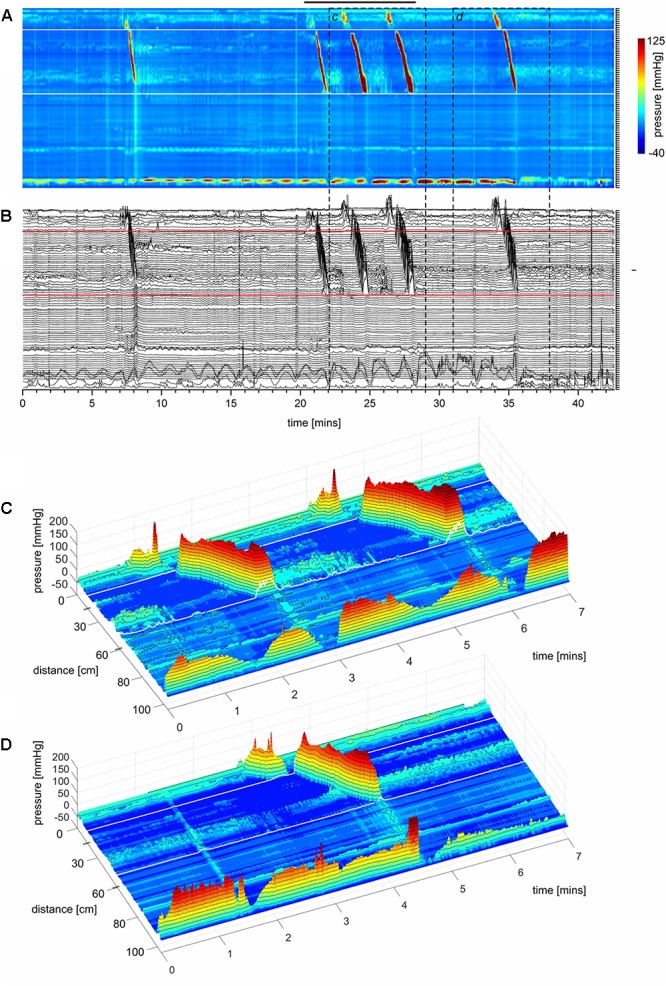
A HAPW that develops into a SPW in response to proximal balloon distention, the HAPW-SPW. In this and all other figure panels, the position of a balloon is indicated by a white line. This means that there are 10 cm gaps in this recording at the two white lines. Hence a recording including 80 sensors will have a total reach of 100 cm. In **(A,B)**, the dashes at the right side of the figure represent the position of the sensors (80 sensors were in the colon, spaced 1 cm apart). The proximal colon is at the top of the figures. In **(C,D)** on the distance axis, 0 is the location of the most proximal sensor. **(A)** The time period of proximal balloon distention by 240 ml air is indicated by the black line above **(A)**. The balloon is positioned at the proximal white line. Proximal HAPWs developed into SPWs (generating HAPW-SPWs) associated with extensive anal sphincter relaxation. Extensive haustral activity (see Chen et al., 2017; Quan et al., 2017) is seen between 30 and 35 cm following each HAPW. **(B)** Same as **(A)**, shown are the actual pressure traces. **(C)** Section of **(A)** between the first two vertical dashed lines show two HAPW-SPWs that are followed by anal sphincter relaxation. Note that the HAPW evoked by the balloon starts at the most proximal sensor or more proximal. **(D)** Section of **(A)** between the 3rd and 4th vertical dashed lines. A low amplitude SPW with anal sphincter relaxation is seen at 1.8 cm. This is followed by an HAPW-SPW and anal sphincter relaxation; the relaxation is preceded by a brief voluntary external anal sphincter contraction. The subject was asked a minute after the motor pattern occurred whether or not the sphincter was voluntarily squeezed. The first HAPW-SPW during balloon distention was associated with bloating, the second with a feeling of urgency. The HAPW-SPW that occurred ∼5 min after balloon distention was associated with urgency. The SPW was not associated with gas or liquid expulsion.

**FIGURE 2:**
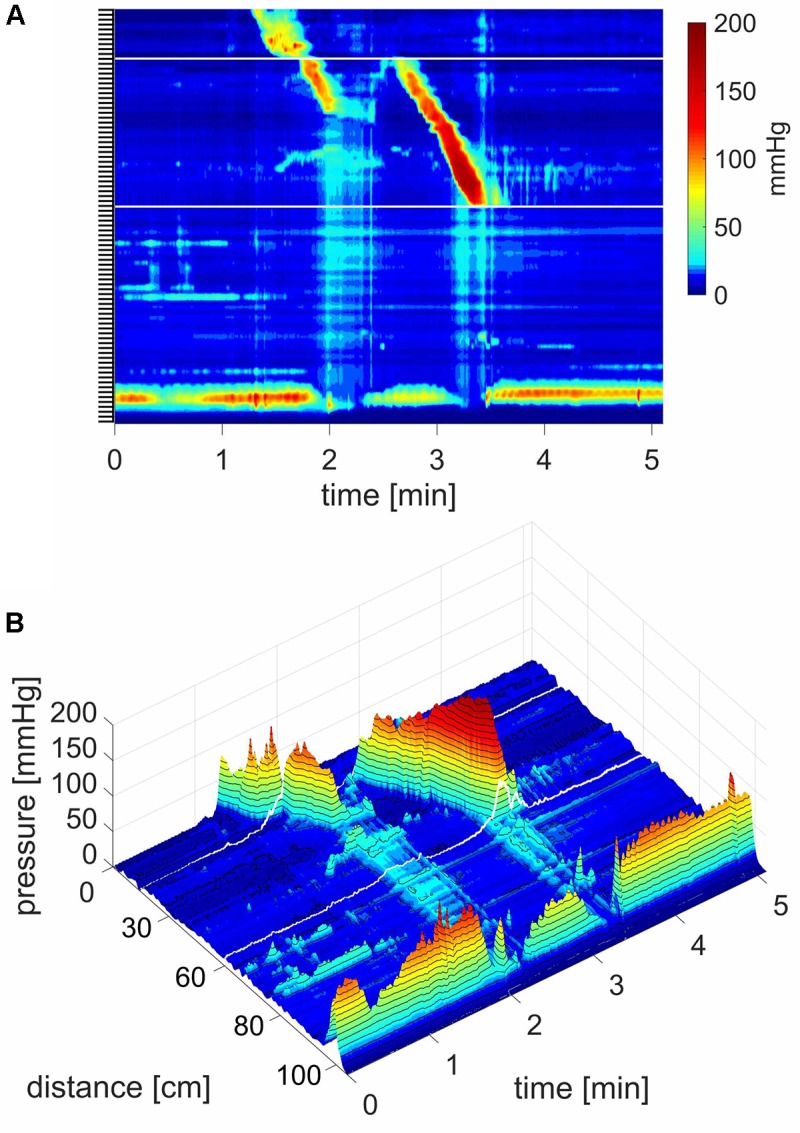
Two proximal High Amplitude Pressure Waves (HAPWs) that develop into SPWs: HAPW-SPWs in response to a meal. **(A)** HAPWs that turn into SPWs with full anal sphincter relaxation. The image shows activity in 100 cm of the colon (position of 84 sensors are shown at the left side of the figure). The activity was observed 12 min after meal intake. The first HAPW-SPW was associated with liquid outflow, the second with liquid and gas expulsion. **(B)** 3D representation of **(A)**.

**FIGURE 3:**
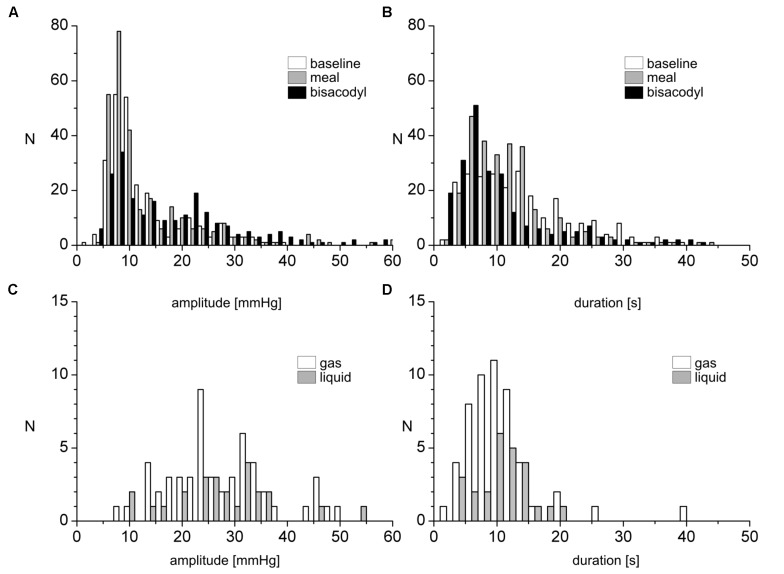
Amplitude and duration distribution of SPWs and their relationship to gas or liquid expulsion. **(A,B)** Number of occurrences of SPWs, binned according to amplitudes **(A)** or duration **(B)**, observed in 17 subjects, before and after a meal and in response to bisacodyl. **(C,D)** Number of occurrences of SPW amplitudes **(C)** and durations **(D)** for SPWs associated with gas or liquid expulsion under all conditions.

Here we report in healthy subjects that SPWs can emerge at the termination of a proximal HAPW (**Figures 1**, **2**), this pattern was termed HAPW-SPWs. At baseline, the SPW component of the HAPW-SPW had an average amplitude of 20.2 ± 10.2 mmHg compared to pancolonic SPWs at 12.1 ± 7.3 (*P* < 0.05), and an average duration of 18.0 ± 12.3 s, associated with a proximal HAPW with an amplitude of 105.4 ± 49.7 mmHg (**Figure 3** and **Table 1**). This study involved 6 females and 11 males, a total of 241 SPWs were observed in females and 547 in males. The total occurrence of SPWs was not significantly different per subject in males (49.7 ± 10.7) vs. females (40.2 ± 5.7) (*P* = 0.539). The average amplitude, however, was significantly lower in females: 12.5 ± 6.3 for females and 17.0 ± 11.0 for males (*P* < 0.0001).

**FIGURE 4:**
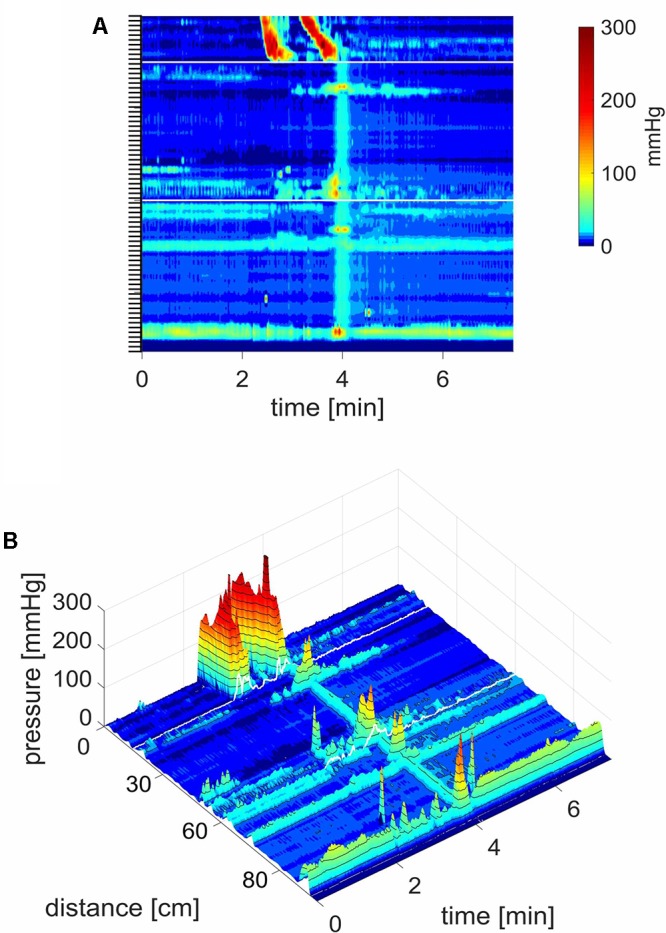
A proximal HAPW and an HAPW-SPW in response to a meal. **(A)** Two very similar proximal HAPWs appeared in response to a meal, but only one developed into an SPW. The SPW was accompanied by external sphincter contraction and by sphincter relaxation and gas expulsion. The SPW started at the end of the HAPW, hence it was not pan-colonic. The first HAPW was not asssociated with gas expulsion. Position of the pressure sensors is shown on the left side. **(B)** 3D rendering of **(A)**. The 0 cm position is in the proximal colon.

Whether or not an HAPW developed into an SPW was not predictable based on the HAPW amplitude. **Figure 4** shows two very similar proximal HAPWs, but only one developed into an SPW.

**FIGURE 5:**
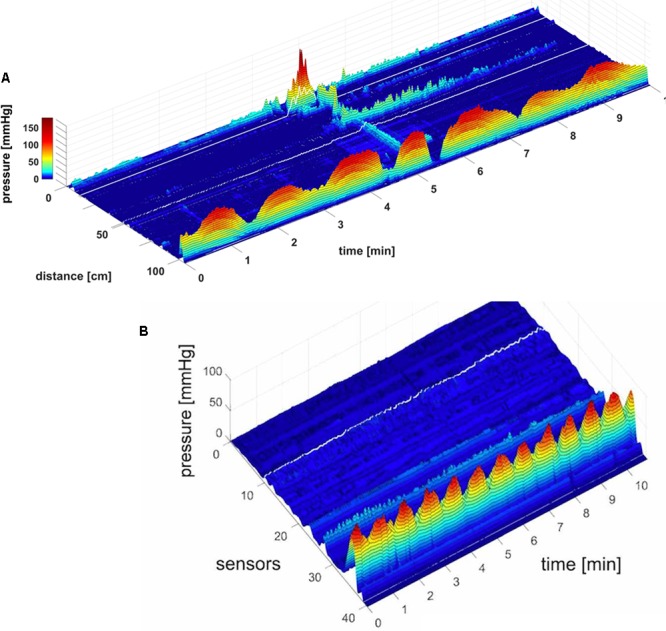
Rhythmic activity of the internal anal sphincter. **(A)** Rhythmic activity of the internal anal sphincter at 0.7 cpm. In addition, a short proximal HAPW appears followed by an SPW and full anal sphincter relaxation. This activity was recorded 80 min after meal intake. **(B)** Low amplitude colonic activity during baseline with rhythmic anal sphincter activity at 1.2 cycles per min.

**FIGURE 6:**
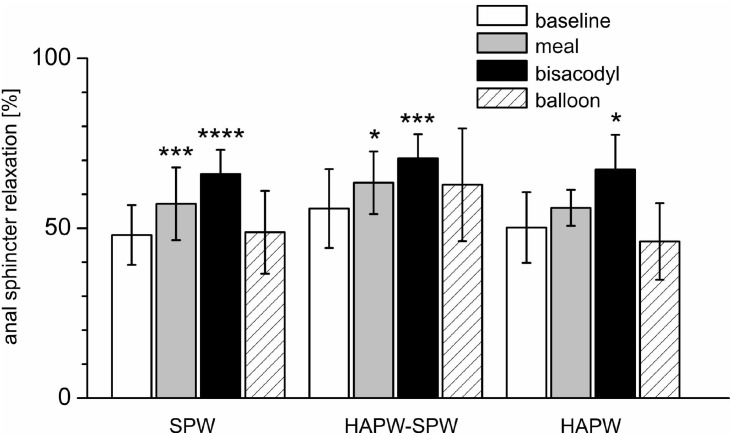
Relaxation of the anal sphincters associated with different motor patterns and in different conditions. Shown is the relaxation of the anal sphincter as a percentage of the anal sphincter pressure just prior to the motor patterns indicated on the X axis. ^∗^*P* < 0.05; ^∗∗∗^*P* < 0.01; ^∗∗∗∗^*P* < 0.001.

The anal sphincter showed intrinsic rhythmicity. In 12 subjects, highly rhythmic internal anal sphincter pressure transients occurred at 1.22 ± 0.26 cpm, oscillating between 62.2 ± 8.2 and 90.5 ± 12.0 mmHg (**Figure 5** and **Table 2**). Because of the spontaneous oscillatory nature of the anal sphincter pressure, relaxation was quantified when it reduced the average pressure value > 25%. Sixty-seven percent of pan-colonic SPWs (124 out of 185) were associated with measurable anal sphincter relaxation (**Table 2**), whereas 88% of HAPW-SPWs were associated with measurable anal sphincter relaxation. Anal sphincter relaxation most often occurred the moment the SPW propagated into the anal canal, but it sometimes started with a delay, sometimes after the SPW ended. Meal and bisacodyl induced higher degrees of SPW-related anal sphincter relaxation (*P* < 0.005, **Figure 6** and **Table 2**) compared to baseline. Combining pan-colonic SPWs and SPWs that followed HAPWs, the anal sphincter relaxed 50.3 ± 9.5% at baseline, which increased to 59.0 ± 10.3% (*P* < 0.01) after a meal and to 69.1 ± 7.1% (*P* < 0.001) in response to bisacodyl (**Table 2**). These are average values of relaxation but in many cases a short period of complete anal sphincter relaxation was achieved (**Figures 1**, **2**). The duration of anal sphincter relaxation was 15.6 ± 5.8 s in baseline, 11.8 ± 4.2 s in the meal session (*P* < 0.01 compared to baseline) and 14.8 ± 6.0 s in the presence of bisacodyl. The average amplitude of SPWs associated with anal sphincter relaxation was 21.0 ± 8.8 mmHg (*n* = 124) compared to 10.0 ± 6.1 mmHg (*n* = 61) without quantifiable anal sphincter relaxation (*P* < 0.01). Nevertheless, the amplitude of SPWs that were associated with anal sphincter relaxation and gas or liquid expulsion spanned the entire repertoire of amplitude and duration (**Figure 3**). When subjects reported gas expulsion, this was always associated with the occurrence of an SPW or an HAPW-SPW. Low amplitude SPWs (<30 mmHg) and associated anal sphincter relaxations were not noticed by the subjects, except when gas or liquid expulsion occurred. Higher amplitude SPWs (>35 mmHg) could be associated with urge and on occasion subjects wanted to prevent outflow and tightened the external anal sphincter as in **Figure 1D**.

**Table 2:** Anal sphincter relaxation associated with SPWs, HAPW-SPWs and HAPWs.

	SPW amplitude (mmHg ± SD)	Anal sphincter amplitude before relaxation (mmHg ± SD)	Anal sphincter amplitude during relaxation (mmHg ± SD)	% of anal sphincter relaxation	Duration of anal sphincter relaxation (seconds)
SPW (*n* = 124), 67% of total (*n* = 185) associated with anal sphincter relaxation					

Baseline (n = 52)	18 ± 7.7	60.2 ± 20.6	31.25 ± 12.5	48.0 ± 8.8	15.6 ± 5.8
Meal (*n* = 44)	21.5 ± 9.5 ns	65.6 ± 19.7	27.52 ± 9.16	57.2 ± 10.7^∗∗∗^	11.8 ± 4.2^∗∗^
Bisacodyl (*n* = 24)	25.3 ± 6.7^∗∗^	75.6 ± 19.9	25.9 ± 8.4	66.0 ± 7.1^∗∗∗∗^	14.8 ± 6.0 ns
Balloon (*n* = 4)	24.5 ± 14.7 ns	76.6 ± 16.2	42.8 ± 22.2	48.8 ± 12.2 ns	12.3 ± 7.0 ns

HAPW-SPW (*n* = 51), 87.9 % of total (*n* = 58) associated with anal sphincter relaxation					

Baseline (*n* = 16)	21.8 ± 6.1	70.8 ± 18.4	31.2 ± 13	55.8 ± 11.6	19.6 ± 7.7
Meal (*n* = 16)	24.4 ± 7.1ns	84.5 ± 29.5	30.3 ± 12.6	63.4 ± 9.2^∗^	18 ± 9.3 ns
Bisacodyl (*n* = 15)	29.4 ± 12 ns	83 ± 25.2	24.3 ± 9.6	70.6 ± 7.1^∗∗∗^	16 ± 7.55 ns
Balloon (*n* = 4)	16.4 ± 16 ns	72.7 ± 6.40	26.6 ± 16	62.8 ± 16.6 ns	21.2 ± 3 ns

HAPW (*n* = 20), 46.5% of total (*n* = 43) associated with anal sphincter relaxation					

Baseline (*n*= 6)	119.4 ± 37.2	72.0 ± 18.6	35.9 ± 12.3	50.2 ± 10.4	20.3 ± 7.5
Meal (*n* = 3)	120.3 ± 16.8 ns	65.7 ± 12.7	29.2 ± 9	56.0 ± 5.3 ns	6.3 ± 4.1^∗^
Bisacodyl (*n* = 8)	207.0 ± 51.0^∗∗^	82.2 ± 11.0	27.2 ± 10.4	67.3 ± 10.2 ^∗^	22.6 ± 10.8 ns
Balloon (*n* = 3)	129.0 ± 33.5 ns	86.6 ± 19.0	29.2 ± 9.0	46.1 ± 11.3 ns	9.6 ± 8.9^∗^


*All compared with their baseline conditions ns P > 0.05; ^∗^P ≤ 0.05; ^∗∗^P ≤ 0.01; ^∗∗∗^P ≤ 0.001; ^∗∗∗∗^P ≤ 0.0001.*

**FIGURE 7:**
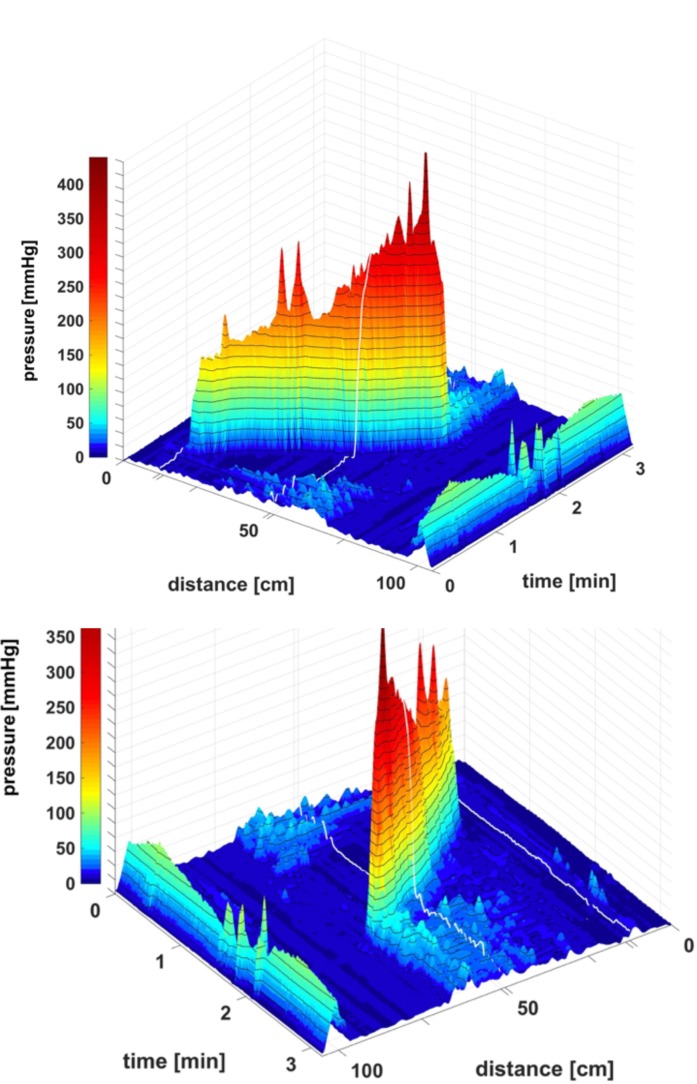
An HAPW that was not followed by an SPW, was not associated with anal sphincter relaxation. Most HAPWs that are associated with anal sphincter relaxation terminate into an SPW that enters the anal canal. In this subject, an HAPW that terminated suddenly and was not followed by an SPW and did not evoke anal sphincter relaxation. This motor pattern occurred 4 min into a proximal balloon distention (240 ml air). The motor pattern started distal to the balloon distention.

An HAPW that was not followed by an SPW, was often not associated with anal sphincter relaxation (**Figure 7**). Anal sphincter relaxation did occur with 47% of HAPWs that were not followed by a SPW (**Table 2**); the relaxation usually started when the HAPW progressed into the sigmoid colon. An HAPW that did not reach the distal colon was rarely associated with anal sphincter relaxation.

#### SPWs in Response to a Meal

**FIGURE 8:**
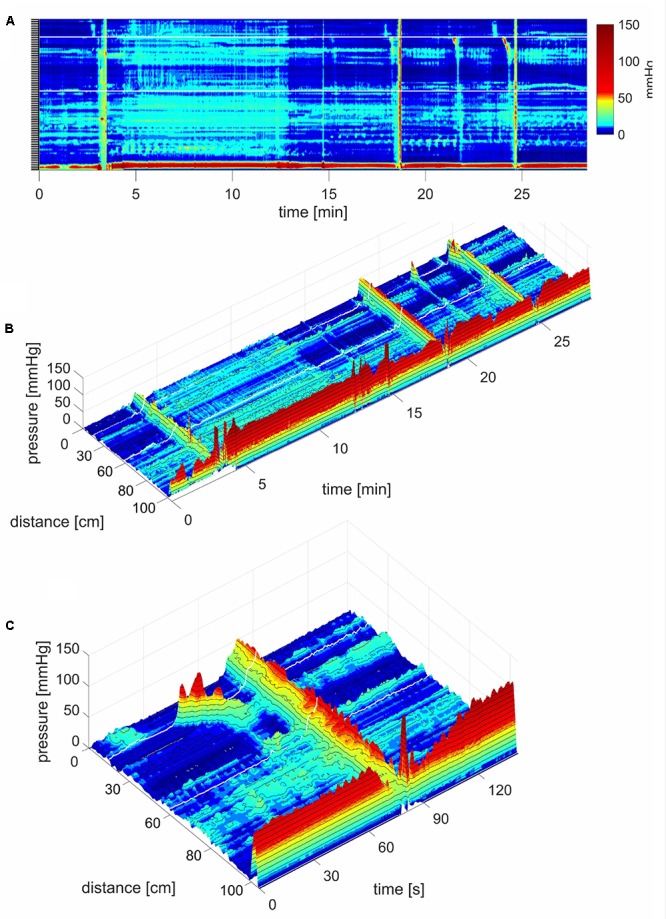
Simultaneous Pressure Waves in response to a meal. **(A)** SPWs that span the entire colon (102 cm). The lighter area, between ∼4 and 12 min, is the time period where the subject sits up causing an increase in intraluminal pressure, and takes in the meal. In this subject, a meal did not induce HAPWs but relatively high amplitude SPWs associated with liquid expulsion. An SPW appeared just before (anticipating the meal) and two following the meal. Anal sphincter relaxations were part of the SPW activity and the SPWs were consistently preceded by relatively low amplitude proximal propagating pressure waves. In addition, a lot of segmenting activity occurred. No pressure waves appeared in the time period between the end of balloon distention and the start of meal intake which was 20 min, except for the one just 1 min before meal intake. All three SPWs were associated with liquid outflow. **(B)** A 3D rendition of **(A)**. **(C)** 3D image of an SPW.

After a 1000 kcal meal, the number of SPWs increased from 3.2 ± 0.5 to 6.4 ± 0.8 (*P* < 0.001) in the first 30 min after a meal compared to that in the last 30 min at baseline (**Figure 8**). The number of gas expulsion-related SPWs increased with an increased average amplitude compared to baseline (29.4 ± 12.6 vs. 15.3 ± 8.4 mmHg, *P* < 0.05, **Table 1**) and compared to SPWs without gas expulsion (12.6 ± 10.3 mmHg, *P* < 0.0001, **Figure 3**). The duration of SPWs did not show significant change (**Table 1**).

### The Rhythmicity of SPWs

**FIGURE 9:**
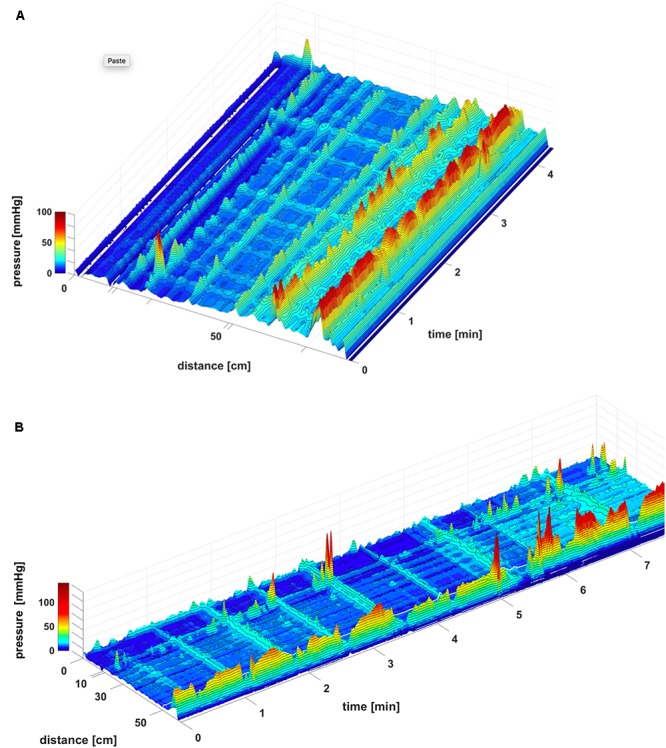
Rhythmic SPWs after meal intake. **(A)** Note that SPWs start at sensor 11, hence not in the proximal colon. The SPWs did not obliterate ongoing haustral boundary activities. *In vivo*, this likely means that under such conditions, gas can pass but stool flow will be restricted by the haustral boundaries. A functional sphincter is prominent, 12 cm above the anal sphincters. The response was observed 44 min after meal intake. There was no gas or liquid outflow. **(B)** Rhythmic SPWs with anal sphincter relaxation. Note that increasing SPW amplitudes are associated with increasing anal sphincter relaxations. This response was observed 150 min after meal intake. There was gas expulsion with the SPW at 3.5 min, and liquid outflow with the SPWs at 5.5 and 7.4 min.

Pan-colonic SPWs occurred in a rhythmic fashion on 26 occasions at an average frequency of 1.77 ± 0.77 cycles/min, ranging from 0.32 to 4.37 cycles/min. Furthermore, 13 of the rhythmic SPW activities were observed in response to the meal where the average frequency was 2.05 ± 0.90 cycles/min (**Figure 9**). **Figure 9B** shows an example where rhythmic SPWs increase in amplitude with time, accompanied by increasing anal sphincter relaxation.

**FIGURE 10:**
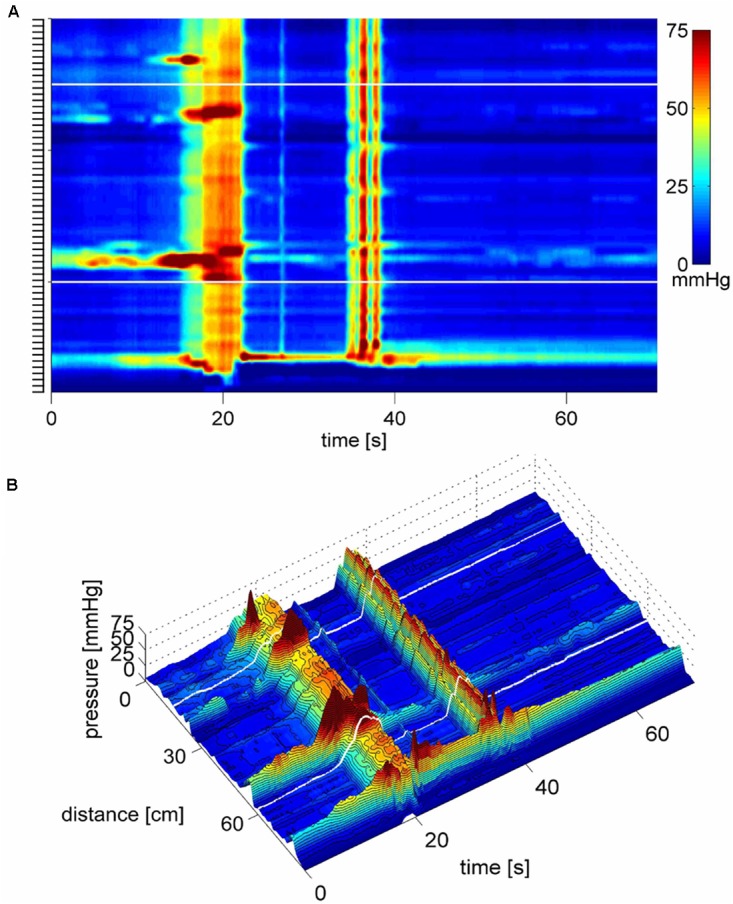
SPWs consist of multiple high frequency pressure waves. **(A)** Two SPWs appear in response to the meal. They can be seen as consisting of clusters of high frequency multiple pressure waves, suggesting that they may be caused by a cluster of rapidly propagating circular muscle contractions as was shown to be the case in the rabbit colon (Quan et al., 2017). **(B)** 3D image of **(A)**.

A second rhythmicity was observed within SPWs. This rhythmicity became apparent when the SPW was not of uniform amplitude. In those instances, SPWs were observed to consist of clusters of very narrow, high frequency SPWs at 24 ± 2 cycles/min (*n* = 5; **Figure 10**).

### SPWs Occurred Independent of Haustral Boundaries and Isolated HAPWs

An often-occurring motor pattern showed irregular or rhythmic pressure transients observed at single censors about 5 cm apart (**Figure 9A**), likely representing haustral boundaries (Chen et al., 2017; Quan et al., 2017). When rhythmic, the dominant frequency was ∼3 cpm. When SPWs traversed these haustral boundary pressure transients, their amplitudes summated, hence SPWs did not abolish the haustral boundaries (**Figure 9A**).

#### SPWs in Response to Proximal Balloon Distension

Proximal balloon distension did not, on average, increase the number of SPWs per unit time compared to baseline, but the number of SPWs associated with gas expulsion markedly increased. Furthermore, the amplitude of SPWs accompanied by gas expulsion was also significantly increased compared to SPWs without gas expulsion (**Table 1**). Proximal balloon distension prominently evoked HAPWs as well as proximal HAPWs that turned into SPWs (**Figure 1** and **Table 1**).

#### SPWs in Response to Bisacodyl Delivered in the Proximal or Descending Colon Through the Catheter

Luminal administration of bisacodyl (10 mg) in the proximal colon or descending colon evoked HAPWs and HAPW-SPWs that started in the proximal colon with associated gas or liquid expulsion; the amplitudes of HAPWs, HAPW-SPWs and SPWs with gas expulsion were higher than that of baseline (**Table 1**). SPWs associated with gas expulsion after meal and bisacodyl had significantly higher amplitudes than those at baseline (*P* < 0.005) but similar duration (**Table 1** and **Figure 3**).

#### SPWs in Response to Rectal Stimulation

**FIGURE 11:**
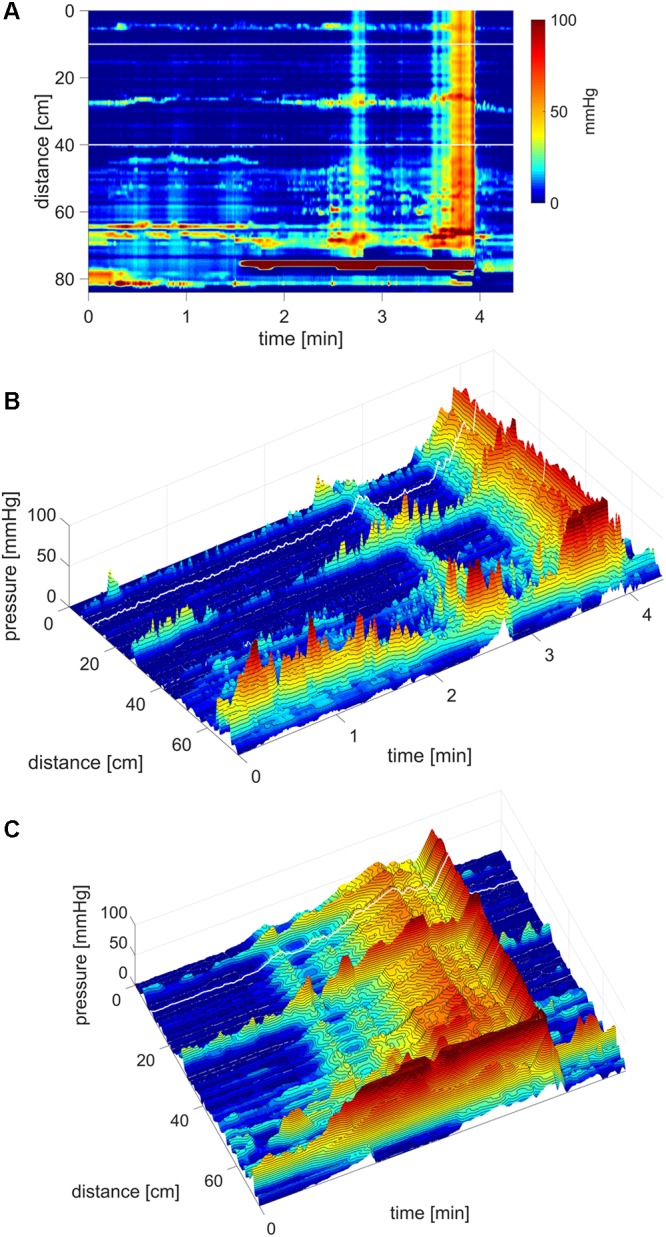
A high amplitude SPW associated with balloon expulsion. **(A)** Two SPWs appear in response to rectal balloon distention; the dark area above the anal sphincter shows the pressure in the rectum induced by the balloon. The first SPW was associated with liquid outflow. The second SPW consists of a cluster of high frequency multiple pressure waves, the last wave at 55 mmHg is followed by relaxation of the sphincter and it expels the balloon. Preceding the balloon expulsion, the SPW was associated with pain and urge. **(B)** 3D image of **(C)**, the balloon distention and anal sphincter activity was removed to make the SPW better visible. **(C)** Close up of the second SPW as shown in **(B)** where its composition of multiple pressure waves is clearly visible.

Rectal balloon distention evoked 10.5 ± 1.2 SPWs in a 20 min period (*N* = 4) vs. 3.5 ± 1.1 in baseline over the same time period (*P* < 0.001) as well as proximal HAPWs followed by SPWs (1.5 ± 1.2 vs. 0.3 ± 0.5; *P* < 0.01)). The average SPW amplitude increased from 5.6 ± 1.6 to 11.1 ± 2.3 mmHg (*P* < 0.05). In **Figure 11**, the SPW is shown to consist of a cluster of high frequency SPWs with the last one reaching an amplitude of 55 mmHg with a strong urge to defecate, followed by expulsion of the balloon.

**FIGURE 12:**
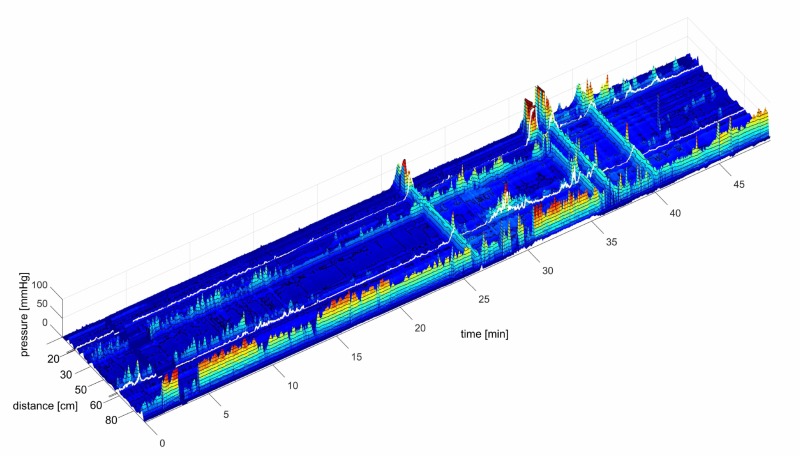
High amplitude SPWs in response to rectal bisacodyl. A 10 mg bisacodyl suspension was injected in the rectum at 16 min. Between 3 and 4 min, data acquisition was halted due to refilling of water reservoirs. This subject produced normal HAPWs in response to balloon distention (not shown). The average amplitude of the SPWs in response to bisacodyl was 32.2 mmHg. All 4 SPWs were associated with liquid outflow and the middle two SPWs with gas expulsion as well.

Rectal bisacodyl (10 mg suspension in water, measured over 30 min periods; *n* = 6) evoked an increase in the number of SPWs: 1.2 ± 0.5 at baseline, compared to 4.8 ± 1.3 after bisacodyl (*N* = 4; *P* < 0.01, at an amplitude of 13.2 ± 5.2. mmHg). HAPW-SPWs increased from 0.2 ± 0.1 during baseline to 4.7 ± 1.2 in the presence of bisacodyl (*P* < 0.001) in 30 min, at an amplitude of 9.5 ± 2.3 mmHg. The effects started 5–8 min after bisacodyl administration (**Figure 12**).

## Discussion

This study shows pan-colonic motor patterns in healthy subjects using an 84-sensor water-perfused catheter that allowed balloon distention and bisacodyl stimulation in the proximal and distal colon. Furthermore, rectal stimulation was assessed.

The present study shows that pan-colonic SPWs, and SPWs that follow proximal high-amplitude propagating pressure waves (HAPW-SPWs), are the dominant propulsive motor patterns observed in HRCM in healthy subjects, since they have a strong association with anal sphincter relaxation and gas and/or liquid expulsion. The SPW amplitudes during baseline and after a meal range from 5 to 61 mmHg and their duration from 2 to 38 s. Meal-induced SPWs indicate that the SPWs are part of the gastro-colonic reflex activity. The strong association of SPWs with anal sphincter relaxation suggests a neurogenic program underlying the motor pattern similar to esophageal contraction associated with lower esophageal sphincter relaxation (Goyal and Paterson, 2011). The observation that SPWs can occur in a highly rhythmic manner, suggests that networks of interstitial cells of Cajal (ICC) might determine part of their pattern (Quan et al., 2017); indicating that neurogenic and myogenic mechanisms overlap (Huizinga and Lammers, 2009; Costa et al., 2013).

**FIGURE 13:**
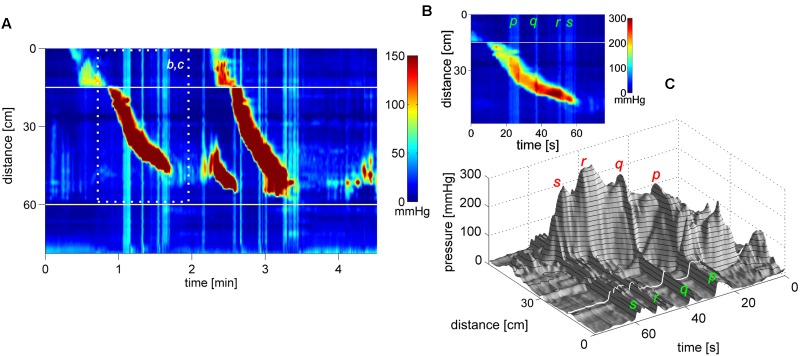
Independent HAPWs and SPWs show addition of pressure. **(A)** Concomittant occurrence of HAPWs and SPWs. The anal sphincter pressure is not shown. **(B)** A closeup of one of the HAPWs with unrelated SPWs. **(C)** The 3D graph of **(A)** shows that the pressures of the HAPW and the SPWs add up. This activity started 8 min after injecting 20 mg bisacodyl suspension in the descending colon. There was no outflow felt.

The number of pancolonic SPWs that were evoked by balloon distention or bisacodyl was likely influenced by the number of HAPWs that were evoked since they did not often occur simultaneously. When they did occur simultaneously, the amplitudes of HAPWs and SPWs summated, further suggesting their independence (**Figure 13**).

In the present study, gas expulsion was always accompanied by an SPW; this suggests that the SPW is a biomarker for gas transit. It was shown that poor handling of gas in IBS patients is a consequence of dysmotility (Serra et al., 2010), and the present study suggests that this may be related to poor sensitivity to evoke SPWs or poor development of SPWs. Bassotti noted that spontaneous gas expulsions were associated with 18 ± 3 mmHg propagating contractions (Bassotti et al., 1996), using an 8-sensor catheter with 12 cm spacing. These contractions were assumed to be similar to mass movements, but the tracings suggest that there was an HAPW in the descending colon that switched to an SPW in the distal colon followed by anal sphincter relaxation; this fits the HAPW-SPW pattern. Mass movements of colonic content involve obliteration of the haustra in the rabbit (Ehrlein et al., 1982) and human (Holzknecht, 1969) allowing smooth passage of stool. The present study shows that SPWs allowed haustral boundary pressure transients to remain (**Figure 9**), which likely allows passage of gas while stool remains undisturbed, which is indeed observed with *in vivo* X-ray imaging in the rabbit (Ehrlein et al., 1982) and in humans where semiliquid content passes over scybala which remain inside the haustra (Ritchie, 1968, 1971; Ritchie et al., 1971). The present study, for the first time, shows that SPWs are associated with liquid outflow, when using a water-perfused catheter. SPWs were seen to reach high amplitudes (>50 mmHg) in response to rectal balloon distention (**Figure 11**) and were associated with urge to defecate and expulsion of the balloon. This suggests that SPWs may be involved in the process of normal defecation, consistent with observations during natural stool evacuation (Bampton et al., 2000). Natural stool evacuation was associated with simultaneous pressurization of the colon, although some or all of the simultaneous pressurizations might have been due to straining. HAPWs occurred well before evacuation and appeared to be associated with moving content into the rectum and urge to defecate (Bampton et al., 2000).

Simultaneous pressure waves were more or less ignored for a long time because pan-colonic pressure changes can be caused by general abdominal pressure increases and in some studies such events were deliberately filtered out (Wiklendt et al., 2013). Indeed, abdominal pressure changes, due to coughing, straining or changing of body position, will give simultaneous pressure changes, but these activities are usually associated with external sphincter contraction, not with anal sphincter relaxation (Chen et al., 2017) and they are associated with abdominal muscular action potentials whereas SPWs are not (Corsetti et al., 2017). It is, of course, critical that these artifacts are recognized and noted during a colon function assessment by HRCM and eliminated from the analysis. Rao et al. (2001) defined SPWs as pressure waves that occurred simultaneously at 3 or more sensors; the sensors were six strain gages with variable distances from 7 to 15 cm. The average amplitude was around 55 mmHg and the duration from 6 to 14 s. They increased upon awakening and after a meal (Rao et al., 2001). Using high-resolution manometry, the first studies suggested the SPWs to occur simultaneously throughout the descending colon (Chen et al., 2017) or to be pancolonic (Corsetti et al., 2017). With high-resolution, many more were identified, in particular SPWs of lower amplitude. Rao et al. (2001) observed on average 5/h, Chen et al. (2017) observed on average 25/h at 14 mmHg and 14 s duration. Corsetti et al. (2017) recorded on average 12/h at 15 mmHg and 23 s duration; SPWs increased during a meal. Simultaneous pressurizations were noted in a pediatric population (4–15 years) associated with constipation but were not found in children who were deemed to have normal colon function (Giorgio et al., 2013). In the present study, SPWs were seen as a prominent normal motor pattern in adults from the age of 20. The SPWs were not necessarily pan-colonic. They often followed a proximal HAPW and they were seen to (Chen et al., 2017) start in the transverse colon (**Figure 9A**) or end proximal to the rectum. The change from an HAPW into a SPW can also be deduced from figures in published low-resolution manometry recordings, although the SPW development was usually not mentioned (e.g., Bassotti et al., 1999) or deemed abnormal (e.g., Di Lorenzo et al., 2002). Our previous HRCM study did not show an increase in SPWs with a meal, in contrast to the present study. This is likely due to the fact that the present study used a 1000 kcal meal whereas our study in China used a 320 kcal meal (Chen et al., 2017).

Wessel et al. (2016) reported a motor pattern in children with intractable constipation that appears similar to SPWs: “long-single propagating motor patterns” which traveled “rapidly” across all of the recording sites that spanned the descending and sigmoid colon. In the legend of their **Figure 3** (Wessel et al., 2016) it is stated that very few appear to propagate in any direction suggesting this pattern to be identical to the SPWs reported here. Their amplitude was also similar at 16.2 ± 8.3 mmHg. This pattern occurred in one child rhythmically at 1.2 cpm, analogous to patterns reported in the present study. A discrepancy is the average propagation velocity which was 2.7 ± 0.7 cm/s preprandially (Wessel et al., 2016). In the present study, SPW propagation velocity cannot be calculated because the SPWs occur simultaneously or are too fast. Reports on “long single propagating motor patterns” observed in healthy *adults* differ from SPWs in the present study in both their preprandial amplitude at 49.7 ± 16.5 mmHg and their propagation velocity at 1.8 ± 1.2 cm/s (Dinning et al., 2014), which is the same as the propagation velocity of HAPWs: 0.4 – 2.8 cm/s (Rao et al., 2004; Liem et al., 2012). A propagating motor pattern with a velocity of ∼2 cm/s at an amplitude of 50 mmHg would be identified by us as a low amplitude propagating pressure wave, similar to an HAPW but of lower force.

The factors that determine the initiation of a proximal HAPW or a proximal SPW are not clear. In a study that had as objective to find out possible relationships between ileal and colonic motor events, it was found that only 9% of cecal colon propagating contractions were clearly linked to an ileal motor event (Dinning et al., 1999); this may indicate that the initiation of the SPW and the HAPW-SPW occurs in the most proximal part of the colon.

Based on a previous study in the rabbit colon, we hypothesize that the motor pattern underlying the SPW is a propulsive motor pattern. In the rabbit colon, SPWs are generated by a cluster of very fast propagating contractions at 15–26 cpm (Quan et al., 2017). Here we show that in the human colon, SPWs are composed of a cluster of multiple narrow SPWs at ∼25 cycles/min. This was visible when the different narrow SPWs had varying amplitudes. It may well be that all SPWs are a composite but that pressure waves of similar amplitude merge to present as a single pressure wave. If SPWs originate from fast propagating circumferential muscle contractions, it will explain their propulsive nature. This notion is supported by the fact that fast propagating contractions are a normal feature of the human colon as shown by X-rays (Ritchie, 1968; Hertz and Newton, 1913) and by the presence of very fast propagating bursts of action potentials recorded intraluminally (Bueno et al., 1980). Importantly, Sarna et al. (1981) observed clusters of electrical oscillations at ∼28 cycles/min when recording from the human colon, *in vivo*, with serosal electrodes, similar to the frequency of the narrow high frequency pressure waves shown in **Figures 10**, **11**.

When the rectum was stimulated by balloon distention or bisacodyl, SPWs appeared which started in the proximal colon; we suggest that this necessarily involved extrinsic autonomic nerves. Evidence for spinal involvement was obtained by Kock et al. (1972) when bisacodyl was administered in the rectum in patients with a bladder substituted by a section of the sigmoid colon, contractions appeared in the substitute bladder. In the rectum, both IMA (intramuscular array)- and IGLE (interganglionic laminar endings)- mechanoreceptors (Spencer et al., 2008) detect tension, stretch or ganglia deformation (Blackshaw and Gebhart, 2002; Brierley et al., 2004; Lynn et al., 2005). Likely similar to the distal colon, a proportion of these mechanosensors may have multiple receptive fields which integrate mechanical and chemical information within the rectum (Berthoud et al., 2001). From the rectum this information will go to the sacral defecation center and from there to several brain stem centers (Stiens et al., 1998; Berthoud et al., 2001) including the locus coeruleus–Barrington’s nucleus complex, the nucleus tractus solitarius (NTS) and the insula (Maggi et al., 1988; Nagano et al., 2004; Martínez et al., 2006; Larsson et al., 2012). From there, the dorsal motor nucleus of the vagus may be activated where vagal nerves may initiate activity in the proximal colon where the signal ends within intramuscular arrays that include intramuscular ICC (Takaki et al., 1987; Wang and Powley, 2000; Komuro, 2006; Wang et al., 2014; Powley et al., 2016; Wei et al., 2017).

High amplitude pressure waves can be associated with anal sphincter relaxation as has been shown in the present and previous studies (Malcolm and Camilleri, 2000). However, in previous studies, the presence of SPWs as a continuation of the HAPWs was not contemplated. In the present study, relaxations associated with HAPWs never occurred prior to the onset of HAPWs. In studies where this was suggested, sensors were not placed in the most proximal colon. It is unlikely that the SPW that follows an HAPW is due to passive pressure build up in the part of the colon distal to the HAPW. First, in more than 50% of the HAPWs studied here, the SPW and anal sphincter relaxation did not occur. Second, passive increase in intraluminal pressure does not generate anal sphincter relaxation but anal sphincter contraction when intraluminal pressure is increased by, for example, body movements or coughing.

The SPW may become a biomarker for evaluating motor patterns that facilitate gas transit, the gastro-colonic reflex and extrinsic neural responses to rectal stimulation, with potential diagnostic value in patients with colonic dysmotility. The complex motor pattern of the SPW associated with anal sphincter relaxation is likely under neuro-myogenic control. Rabbit colon experiments have shown us that the SPW is generated by fast propagating contractions which are myogenic and likely controlled by interstitial cells of Cajal associated with the myenteric plexus (ICC-MP) (Lentle et al., 2008) but the organization of these fast propagating contractions into rhythmic clusters is neurogenic (Chen et al., 2016). The situation may be the same in the human colon which has a rich network of ICC-MP (Rumessen et al., 2009) and fast propagation can easily be facilitated by an ICC network (Wei et al., 2017). The SPW that is initiated by a meal or rectal stimulation will have an extrinsic neural origin and the progression along the colon will involve the enteric nervous system and may involve ICC networks.

What is involved in anal sphincter relaxation associated with the SPW? The anal sphincter tone is primarily generated by the internal anal sphincter musculature (Rattan, 2005) and controlled by slow wave activity of ICC-IM that is conducted into internal anal sphincter smooth muscle cells. This slow wave activity is responsible for orchestrating the rhythmic contractile activity (Cobine et al., 2017), which occurs at ∼3 cpm shown in the present study. Autonomic nerves are supplying the internal anal sphincter via the inferior rectal branches of the pelvic plexus (Kinugasa et al., 2014) with sympathetic nerves being dominant. Although sympathetic innervation may not significantly contribute to basal tone, it can cause anal sphincter contraction through direct innervation of smooth muscle cells via alpha 1 receptors (Frenckner and Ihre, 1976; Glavind et al., 1997; Rattan, 2005). The external sphincter makes an important contribution to anal sphincter tone and the mechanism of continence, in part by a sacral reflex (Broens et al., 2013). The relaxation of the anal sphincter is undoubtedly neurogenic. The relaxation of the anal sphincter in response to an SPW can be complete, resulting in the absence of any pressure gradient between rectum and the external environment. This will involve inhibition of internal anal sphincter pressure by enteric nitrergic nerves (De Lorijn et al., 2005) with the sensory arm of the rectal reflex that causes anal sphincter relaxation mediated by ICC (De Lorijn et al., 2005). Inhibition of the internal anal sphincter is also facilitated by activation of inhibitory nerves with VIP or carbon monoxide as transmitter (Rattan, 2005). Inhibition may further be facilitated by parasympathetic activation of myenteric inhibitory nerves to the internal anal sphincter (Gonella et al., 1987). This is supported by studies in the canine where colonic distention decreased pressure in the anal canal via sympathetic pathways (Chen et al., 2010). Relaxation of the external anal sphincter can only be achieved through decrease in the discharge frequency of sacral motor neurons to the external anal sphincter that contribute to anal tone (Gonella et al., 1987). Cell bodies of the pudendal nerve fibers to the external sphincter are in the Onuf’s nucleus and these nerves can be affected by parasympathetic nerves, likely via interneurons, from the sacral defecation center (Roppolo et al., 1985; Kihira et al., 1997).

In summary, SPWs are a prominent colonic-rectal motor pattern, propagating into the anal canal, associated with anal sphincter relaxation and gas expulsion in healthy subjects observed during HRCM. SPWs are also associated with expulsion of liquid and a high amplitude SPW can expel a balloon, hence may be associated with stool expulsion. We report here that SPWs can be initiated at the termination of a proximal HAPW. SPWs can also start in the transverse colon without being preceded by an HAPW. SPWs can be used to assess motor responses to stimuli such as a meal, balloon distention or bisacodyl. HRCM can reveal the ability of the colonic musculature to generate many distinct motor patterns likely associated with distinct functions. HRCM can show intactness or absence of motor patterns associated with neural reflexes in response to distention, a meal or chemical stimuli. It is essential to include SPWs in a clinical assessment of potential colon motor dysfunction.

## Ethics Statement

This study was carried out in accordance with the recommendations of the Hamilton Integrated Research Ethics Board. The protocol was approved by the Hamilton Integrated Research Ethics Board. All subjects gave written informed consent in accordance with the Declaration of Helsinki.

## Author Contributions

J-HC and JH: study concept and design, acquisition of data, analysis and interpretation of data, drafting of the manuscript, critical revision of the manuscript for important intellectual content, and study supervision. SP: design of data analysis, analysis and interpretation of data, critical revision of the manuscript for important intellectual content, and statistical analysis. MS, MX, AW, AE, and KZ: analysis and interpretation of data, critical revision of the manuscript for important intellectual content, and statistical analysis. AV, YY, WC, and MP: acquisition of data, analysis and interpretation of data, and critical revision of the manuscript for important intellectual content. DA, PB, and EG: acquisition of data, critical revision of the manuscript for important intellectual content, and material support. PM and ER: critical revision of the manuscript for important intellectual content, statistical analysis, and obtained funding.

## Funding and Acknowledgments

JH received a Canadian Foundation for Innovation John Evans Leadership grant for the equipment used in this study. Operating funds were obtained from the Hamilton Academic Health Sciences Organization (HAHSO) to ER and from the Canadian Institutes of Health Research (CIHR) to JH. The Farncombe Family Digestive Health Research Institute provided partial salary support for J-HC, SP, and AV. MS received an Ontario Graduate Scholarship. YY was supported by Sun Yat-sen University Hospital. MX received an NSERC summer student scholarship. The authors want to acknowledge Prof. Stephen Collins for supporting HRCM at McMaster University. The hardware was designed in collaboration with Medical Measurement Systems. The catheters were designed in collaboration with Howard Mui and staff at Mui Scientific. Parts of this research were presented at the Canadian Digestive Diseases Week of 2018 (Chen et al., 2018).

## Conflict of Interest Statement

The authors declare that the research was conducted in the absence of any commercial or financial relationships that could be construed as a potential conflict of interest.

## Abbreviations

HAPW: high-amplitude propagating pressure wave
HAPW-SPW: a combination of a proximal HAPW followed by an SPW
HRCM: high-resolution colonic manometry
SPW: Simultaneous pressure wave
